# Bronchial artery embolization combined with left pulmonary resection in the treatment of fibrosing mediastinitis complicated with massive hemoptysis: a case report

**DOI:** 10.3389/fmed.2024.1418105

**Published:** 2024-09-26

**Authors:** Min Liu, Jixiang Liu, Shi Chen, Xiaoyan Gao, Lu Sun, Fajiu Li, Chenghong Li

**Affiliations:** ^1^Department of Pulmonary and Critical Care Medicine, Affiliated Hospital of Jianghan University, Wuhan, China; ^2^National Center for Respiratory Medicine, State Key Laboratory of Respiratory Health and Multimorbidity, National Clinical Research Center for Respiratory Diseases, Institute of Respiratory Medicine, Chinese Academy of Medical Sciences, Department of Pulmonary and Critical Care Medicine, Center of Respiratory Medicine, China-Japan Friendship Hospital, Beijing, China; ^3^Institute of Pulmonary Vascular Diseases, Jianghan University, Wuhan, China

**Keywords:** fibrosing mediastinitis, massive hemoptysis, surgical intervention, multimodal therapy, case report

## Abstract

Fibrosing mediastinitis (FM) is a rare and benign fibroproliferative disease that presents with the proliferation of extensive, dense fibrous tissue in the mediastinum. Hemoptysis is a common clinical manifestation of FM. Clinically, most patients exhibit mild to moderate hemoptysis. We report a case of FM complicated with life-threatening massive hemoptysis. The patient was successfully rescued through a combination of bronchoscopic balloon closure, bronchial artery embolization (BAE), and surgical interventions. Although FM is frequently benign, vascular involvement can progress to life-threatening massive hemoptysis and must be treated appropriately.

## Introduction

Fibrosing mediastinitis (FM) is a rare and benign fibroproliferative disease in the mediastinum (1). Proliferative fibrous tissue gradually replaces normal fat tissue and wraps, infiltrates, and compresses the adjacent structures in the mediastinum, such as pulmonary vessels, superior vena cava (SVC), bronchus, esophagus, and pericardium (2). The aberrant behavior of the proliferative fibrous tissue may cause PH, SVC syndrome, atelectasis, and obstructive pneumonia (3). The clinical symptoms of FM depend on the involved structures in the mediastinum. Specifically, pulmonary vascular compression may lead to severe hemoptysis and even death as a result of systemic collateral hyperplasia, which brings a great challenge to clinical treatment (4).

The pathogenesis of FM is still unclear, and there is no guidance or expert consensus has been established. The efficacy of drug therapy is limited and uncertain (5, 6). Endovascular interventional modality and surgical interventions to relieve mediastinal compression have demonstrated highly variable results (7). Therefore, palliation of symptoms may require a combination of multimodal treatments, including medical, interventional and surgical approaches in selective cases. Herein, we report a case of FM with recurrent life-threatening massive hemoptysis and successfully rescued through a combination of multimodal approaches. We hope this case highlights the difficulty in the treatment of FM with massive hemoptysis and provides insight into the challenges in management strategies.

## Case description

**Figure 1:**
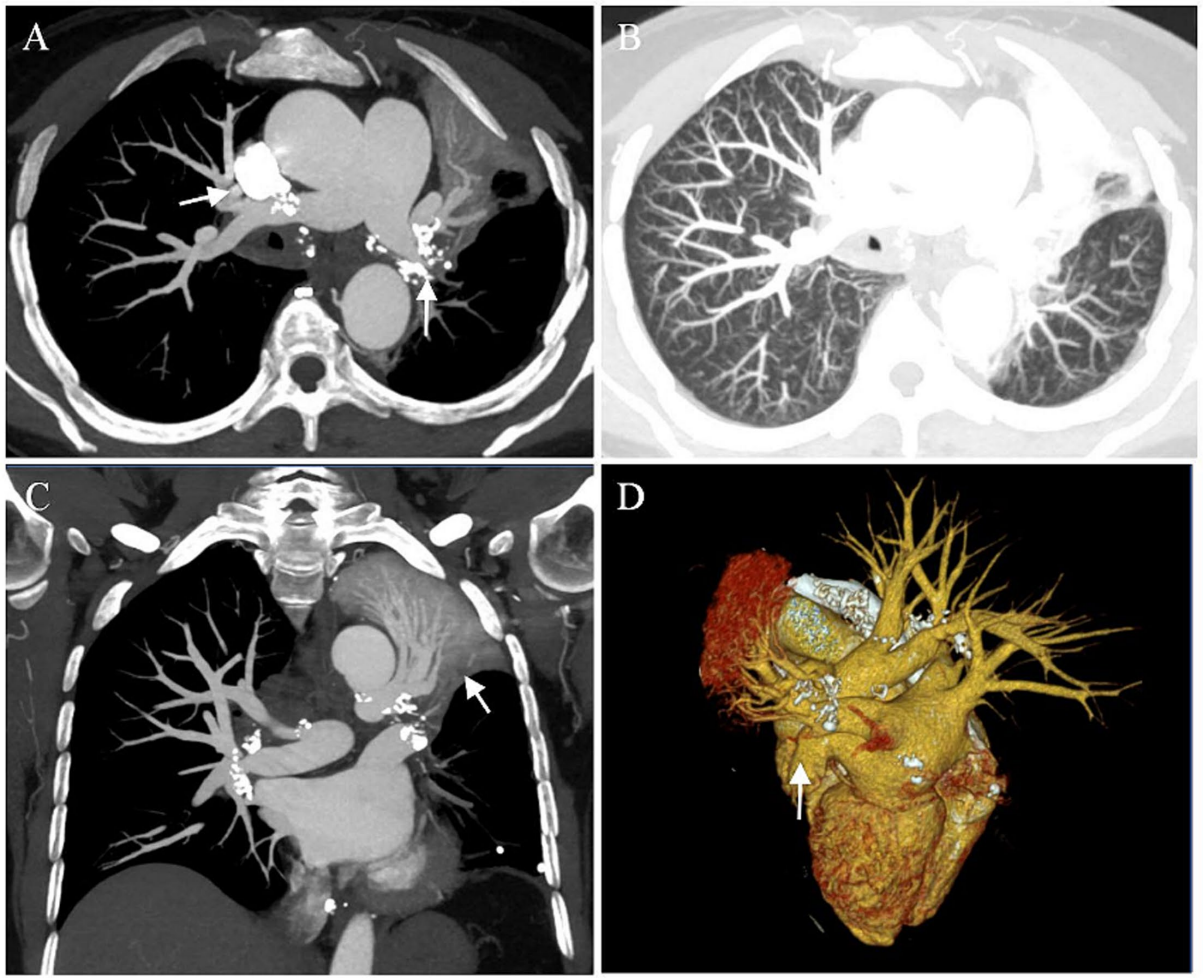
Preoperative images. **(A–C)** Chest CT angiography showed atelectasis of the left upper lung, multiple calcifications of the hilum, occlusion of the left lower pulmonary artery and vein (arrows). **(D)** 3D reconstruction demonstrated arteriovenous occlusion of the left lower lobe (arrow).

A 55-year-old female was transferred to our hospital for hemoptysis. One week ago, she underwent endotracheal intubation and emergency bronchial artery embolization (BAE) in a local hospital due to massive hemoptysis, with an estimated blood loss of 500 mL/day. There was still active bleeding after the interventional operation. For further diagnosis and treatment, she was referred to our center. The patient had a history of hypertension and coronary heart disease. She was a non-smoker and denied alcohol consumption, illicit drug use, and any home or occupational exposures. There was no family history of respiratory diseases. On arrival, she was undergoing assisted ventilation and endotracheal intubation. Chest auscultation was notable for diminished breath sounds and dullness to percussion over the left lung. No heart murmur was appreciated. Laboratory data showed a white blood cell count of 10.60 × 10^9^/L, hemoglobin of 84 g/L, platelet count of 424 × 10^9^/L, ESR of 80 mm/h, high sensitive C-reactive protein of 18.24 mg/L. N terminal pro-B-type natriuretic peptide (NT-proBNP) and coagulation function were mostly normal, except for an elevated fibrinogen (5.47 g/L). Chest CT angiography showed that soft tissue extended throughout the mediastinum towards the hilum with calcification and compressed the adjacent structures, resulting in arteriovenous occlusion of the left lower lobe and atelectasis of the left upper lobe (Figure 1).

**Figure 2:**
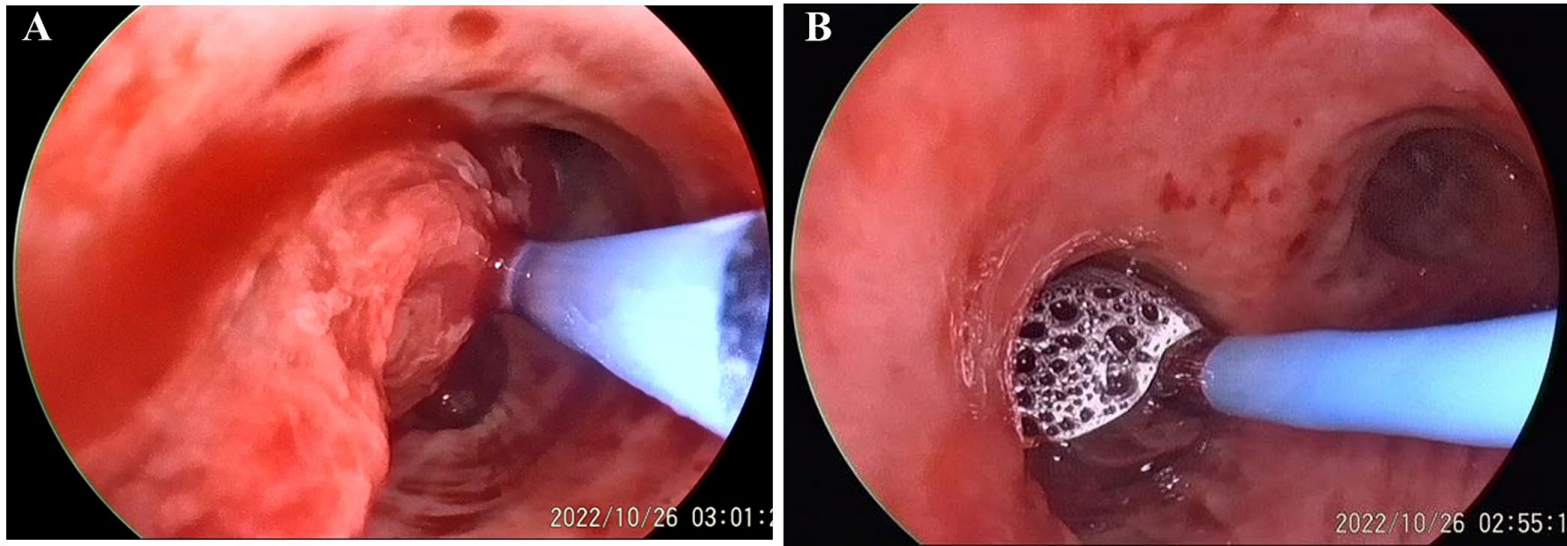
Preoperative bronchoscopy. **(A)** Massive blood clots and active bleeding in the left upper lobe. Cryotherapy cleared the blood clots. **(B)** The left main bronchus is prepositioned with a balloon.

**Figure 3:**
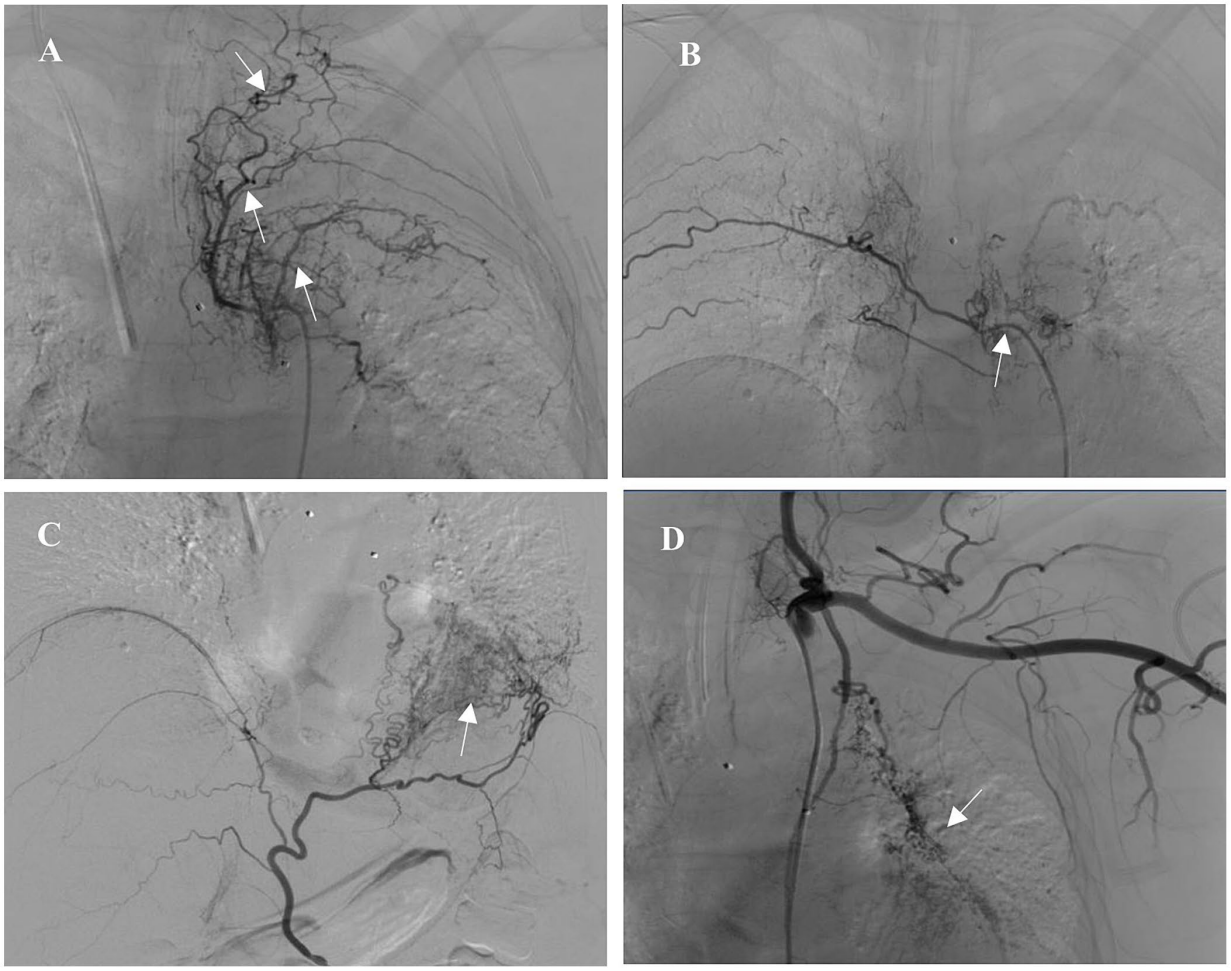
Selective bronchial arteriography revealed systemic collateral hyperplasia, forming a disordered vessel network. **(A)** Left intercostal artery (arrow). **(B)** Right intercostal artery (arrow). **(C)** Left inferior phrenic artery (arrow). **(D)** Left internal thoracic artery (arrow).

After admission, the patient was treated with intravenous piperacillin-tazobactam and hemostatic medicine (hemocoagulase and tranexamic acid). There was no significant improvement in active hemoptysis, approximately several tablespoons of bright red blood every hour. She suddenly experienced a recurrence of severe hemoptysis with massive blood clots almost totally occluding the left main bronchus during the therapy. Bedside bronchoscopic cryotherapy was performed immediately to eliminate the blood clots. Simultaneously, a three-stage balloon dilating catheter (12–13.5–15 mm) was inserted into the left main bronchus, and the balloon was gradually expanded to 13.5 mm (Figure 2). Emergent BAE was subsequently performed. Selective bronchial arteriography revealed compensatory hyperplasia of the systemic collateral vessels, forming a disordered capillary network (Figure 3). According to the pathological vessel diameter, PVA (Cook, United States) with a diameter of 300–1,000 μm was selected to embolize the peripheral vascular bed, and gelatin sponge (GS, Hangzhou Alicon) with a diameter of 150–1,400 μm was used to embolize the main vessel of the responsible vessel. To prevent potential spinal cord ischemia, the branch of intercostal artery supplying the left hilum was not embolized (Figure 3B). Pulmonary angiography was performed and confirmed stenosis of multiple segments of pulmonary artery (PA), and occlusion of the left lower PA. Right heart catheterization demonstrated pulmonary artery pressure systolic/diastolic/mean was 38/13/22 mmHg, pulmonary vascular resistance (PVR) was 2.43 Wood units. Considering that it is difficult to place a stent in an occluded vessel, endovascular intervention was not performed. Postoperative bronchoscopy confirmed no active bleeding. The balloon catheter and tracheal intubation were successfully removed. The patient was discharged after 1 week of anti-infection and hemostatic treatment.

**Figure 4:**
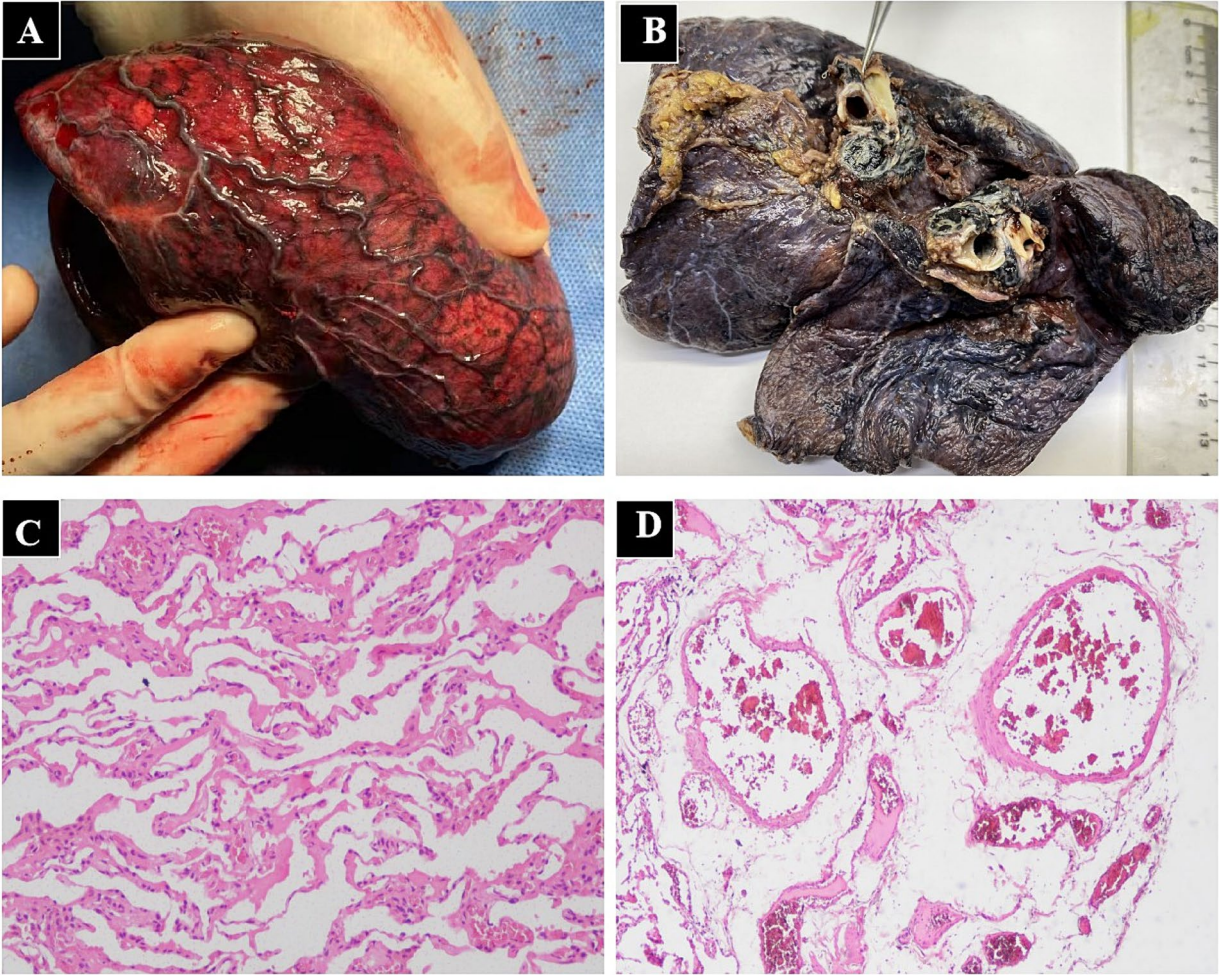
Gross examination and histopathology of lung resection tissue in patients with fibrosing mediastinitis. **(A)** The dilated blood vessels on the surface of the left lower lobe. **(B)** The significant fibrosis of the hilar tissue. Lung histopathology showed pulmonary interstitial hemorrhage with thickening and dilatation of pulmonary vascular, including pre-capillary arteries, pulmonary venules, capillaries **(C)** and subpleural veins **(D)**.

Three days after discharge, the patient had recurrent hemoptysis with a volume of approximately 600 mL and was readmitted to our hospital. After a multidisciplinary discussion, the patient was transferred to the thoracic surgery department and underwent a left pneumonectomy. During the operation, fibrosis involving the mediastinum and hilar structures was observed, accompanied by extensive calcification and multiple enlarged lymph nodes. The irregular, tortuous vessels were found in the mediastinum. After the difficult separation of adhesive mediastinal and hilar tissues, left pneumonectomy was successfully performed. Gross examination manifested that the hilum is black and hard, with dilated vessels on the surface of the left lower lobe (Figures 4A,B). Histopathology showed hilar interstitial fibrosis with calcification, pulmonary interstitial hemorrhage, vascular wall thickening, and dilatation (Figures 4C,D). Immunohistochemistry identified CD3 (scattered +), CD20 (−), PCK (−), Muc5AC (−), KI67 (+5%). An extensive detection for infectious, neoplastic, granulomatous, and autoimmune causes of FM was negative. Therefore, the patient was diagnosed with idiopathic FM. Oxygenation improved significantly after surgery. She remained well and free of complications during 1 year of follow-up.

## Discussion

FM is a rare disease characterized by hyperplasia of fibrous tissue in the mediastinum. FM is often caused by histoplasmosis capsulatum, tuberculosis, sarcoidosis, and autoimmune diseases (8, 9). The clinical manifestations of FM are nonspecific, and common symptoms include dyspnea, hemoptysis, chest pain, and superior vena cava syndrome (SVCS) (10–12). This report described a case of FM with recurrent life-threatening massive hemoptysis. Since no specific cause was identified in this patient, idiopathic FM was a diagnosis of exclusion.

In general, hemoptysis is a common symptom of FM. Mediastinal fibrosis can compress pulmonary vessels and cause external stenosis or even occlusion of pulmonary arteries and veins, resulting in pulmonary hypertension (PH) and systemic collateral hyperplasia, which further led to moderate to severe or even massive hemoptysis (13). In this case, the mediastinal fibrous tissue compressed the adjacent blood vessels and bronchus, resulting in stenosis of the bronchial opening in the left upper lobe and occlusion of the arteriovenous opening in the left lower lobe. It further led to atelectasis of the left upper lobe and systemic collateral hyperplasia. Inadequate blood supply and repeated chronic infection further aggravate the vasculopathy and promote the rupture of the vessel in response to the triggering factors. We demonstrated these secondary vascular changes through the multimodal presentation of imaging, tissue specimens, and pathology. PH and subsequent right heart failure were serious complications and the most common causes of death in FM (14). The main manifestations of this case were collateral vessel hyperplasia and massive hemoptysis, without pulmonary hypertension and right heart dysfunction. It is possible that the compensatory dilatation of the bronchial arteries and recurrent massive hemoptysis may reduce the volume of blood in the pulmonary circulation. Therefore, the severity of FM cannot be assessed only based on pulmonary artery pressure.

Clinical presentation and noninvasive imaging findings can indicate the diagnosis. Imaging is crucial for the assessment of progression, identification of structural compromise, and evaluation of treatment response (7, 15). Pathology is the gold standard for diagnosis of FM and a critical approach to rule out malignancy. Pathological analysis reported nodular or diffuse proliferative fibrous tissue surrounding and replacing mediastinal structures with lymphocyte infiltration. Based on the histopathologic evaluation, a recommended three-stage staging system has been proposed and facilitates treatment options and clinical follow-up (16). Previous histopathological evaluations focused on changes in the components of the disease at different stages of progression. In this case, we investigated the vascular pathologic features and noted that the vascular wall was markedly thickened. It suggested that the vascular lesions caused by FM were not only stenosis or occlusion caused by physical external pressure. Changes in hemodynamics and the microenvironment may further induce secondary microvasculopathy. Further study needs to be conducted to provide more evidence and elucidate mechanisms.

Currently, there is no guidance on the management of FM. Medication, endovascular intervention, and surgery may be indicated for prognostic benefit. Antibiotics, antifungals, and steroids have been described in case series and small retrospective studies (17, 18). Due to the high recurrence rate and mortality associated with surgical treatment, balloon angioplasty or stenting has become the preferred method for the treatment of pulmonary vascular stenosis in recent years (19, 20). This case was in stage III according to the recommended pathological stage and medical treatment was inefficacy and steroids were ineffective in reversing the fibrosing process. Due to pulmonary arteriovenous occlusion, balloon angioplasty and stent implantation were difficult to operate and had high risk. Given that recurrent hemoptysis is the foremost problem in this case, endotracheal intubation, endoscopic balloon dilatation, and BAE may be the best option for a life-saving resuscitation. Recurrence of hemoptysis was due to the inability to embolize certain vessels during BAE, as well as vascular compensation. In such instances, surgery was considered as the most appropriate option. The extensive fibrosis is often dense and rigid, making dissociation extremely difficult. So, fibrosing mediastinis is a very challenging condition for thoracic surgery, even requiring intrapericardial access or cardiopulmonary bypass (21, 22). In addition, oxygenation of this case improved significantly due to the elimination of the severe imbalance between ventilation and blood flow in the left lung. Because of the limited sample sizes, the prognosis of FM patients is not clear. Seferian et al. (23) followed up 27 patients with FM complicating pulmonary hypertension and found that survival rates at 1, 3, and 5 years postdiagnosis were 88, 73, and 56%, respectively. The clinical benefits of surgery have also revealed highly variable results. Peikert et al. (24) have shown that 42% of FM patients subjected to surgical treatment relapse during the follow-up and require other interventions. This patient remained asymptomatic during the follow-up. The long-term efficacy and safety still need to be further evaluated and followed.

In conclusion, FM is a very rare disease that can be life-threatening in severe cases. We reported a case of FM complicated with massive hemoptysis and successfully rescued through a combination of bronchoscopic balloon closure, catheter-based interventions, and surgical treatment. It demonstrated the value of multimodal therapies in selective cases.

## Data Availability

The original contributions presented in the study are included in the article/supplementary material, further inquiries can be directed to the corresponding author.
